# Novel Recombinant Rabbit Hemorrhagic Disease Virus 2 (RHDV2) is Circulating in China within 12 Months after Original RHDV2 Arrival

**DOI:** 10.1155/2023/4787785

**Published:** 2023-08-30

**Authors:** Bo Hu, Zhiyu Fan, Rulong Qiu, Mengmeng Chen, Houjun Wei, Yanhua Song, Weilong Liu, Weizhong Xu, Fang Wang

**Affiliations:** ^1^Institute of Veterinary Medicine, Jiangsu Academy of Agricultural Sciences, Key Laboratory of Veterinary Biologicals Engineering and Technology, Ministry of Agriculture, National Center for Engineering Research of Veterinary Bio-Products, Nanjing, China; ^2^Xizang Agriculture and Animal Husbandry College, Linzhi, China

## Abstract

Rabbit hemorrhagic disease (RHD) causes lethal fulminant hepatitis in rabbits. Two different genotypes (GI.1 and GI.2) responsible for RHD are reported. GI.2 was first detected in France in 2010 and subsequently spread to other countries in Europe. In April 2020, GI.2 was detected in China. In this study, we report a novel recombinant strain of fatal rabbit hemorrhagic disease virus 2 (RHDV2 or GI.2) detected from domestic rabbits in three provinces in China in 2020–2021. Full-length genomic analysis has revealed that the recombinant virus contained an RHDV2 capsid gene and nonstructural genes from an unclassified lagovirus genotype. This type of virus emerged and circulated throughout China within a year after the initial detection of the original RHDV2. Compared with the original strain, the new virus showed a longer infected time and lower mortality rate but almost the same viral load at the moribund stage of infection. This might have resulted in high virus contamination in the environment, facilitating virus transmission. As the consequences of the presence of novel recombinant strains are unpredictable, the circulation of the novel variant in the population should be carefully monitored in China.

## 1. Introduction

**Figure 1:**
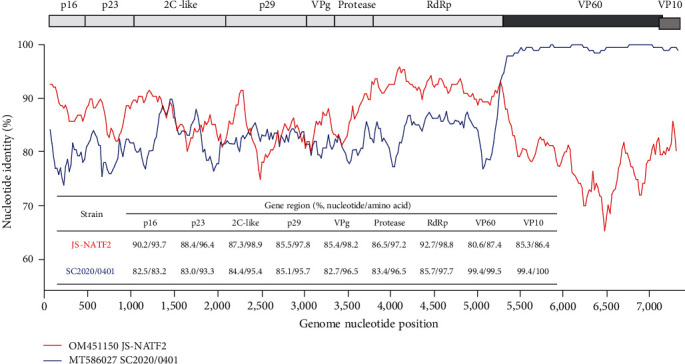
Genome recombination analysis of the SC2020/1016-02 strain. SimPlot software (version 3.5.1) was used for analyzing sequence identity between the SC2020/1016-02 consensus genome and the most likely donor strains JS-NATF2 and SC2020/0401. The nucleotide and amino acid sequence identities of different gene regions in the SC2020/1016-02 strain compared with the JS-NATF2 and SC2020/0401 strains were also analyzed. The vertical axis represents the percentage of sequence identity, and the horizontal axis indicates the nucleotide positions.

Rabbit hemorrhagic disease virus (RHDV), a single-stranded positive-sense RNA virus of the family *Caliciviridae*, genus *Lagovirus*, is associated with high morbidity and mortality in rabbits. RHDV infection causes severe economic losses in rabbit industries and affects wild rabbits and their predators in the ecosystem. Lagoviruses are divided into two genogroups (GI and GII), and the GI genogroup is further subdivided into four variants (GI.1, GI.2, GI.3, and GI.4) [1]. GI.1 contains the classical RHDV (GI.1b–GI.1d) and the antigenic variant RHDVa (GI.1a), while GI.2 represents the more recently discovered pathogenic lagovirus RHDV2 [1, 2]. GI.2 was first detected in France in 2010 [2] and subsequently spread worldwide [3–5]. GI.3 and GI.4 have been characterized as nonpathogenic lagoviruses [1]. All lagoviruses share a similar genomic pattern comprising two open reading frames (ORFs). ORF1 encodes a single polyprotein, which is cleaved into seven nonstructural proteins (p16, p23, 2C-like, p29, VPg, protease, and RdRp), and the major capsid protein VP60, whereas ORF2 encodes a minor structural protein, VP10 (Figure 1).

In China, the prevalent RHDV strains are GI.1a, GI.1c, and GI.2 [6, 7]. The first incidence of GI.2 infection in China was reported in Sichuan province in April 2020 [7], and the virus has subsequently spread to several rabbit farms in other provinces. Although the mechanisms of RHDV emergence have not been clearly identified, previous studies suggest that recombination may play a central role in the emergence and evolution of pathogenic RHDV [8–10]. Several recombination events have been reported in previous studies, particularly for the GI.2, involving other pathogenic (such as GI.1a and GI.1b) or nonpathogenic (such as GI.4) lagoviruses [8, 11–13]. In fact, the first GI.2 strain identified was a recombinant GI.3–GI.2 strain, with the GI.3 strain serving as the major donor for the nonstructural part of the recombinant, whereas the GI.2 strain contributing to the structural part [11, 13]. Here, we report the identification and characterization of a novel recombinant RHDV2 variant, which caused acute fatal disease in domestic rabbits in China.

## 2. Materials and Methods

Liver samples collected from 37 separate RHDV outbreaks (more than 100,000 rabbits were affected in total) in nine provinces in China between 2020 and 2021 were frozen and stored at −70°C. The hemagglutination test was carried out [14], and the positive samples were collected for further analysis. The full-length genome sequences were obtained by the amplification of several overlapping fragments using real-time PCR (RT-PCR), as previously described [8]. Web BLAST (https://blast.ncbi.nlm.nih.gov/Blast.cgi) was used for sequence analysis. Recombination events were screened using Recombination Detection Program (RDP; version 5.5) [15] and SimPlot (version 3.5.1) [16]. Phylogenetic analysis of genome sequences based on the structural part (nucleotides 5,305–7,378) and the nonstructural part (nucleotides 10–5,304) was performed using MEGA 7 (version 7.0.26) [17] with the maximum-likelihood approach based on the general time reversible model with a gamma distribution and invariant sites (GTR + G + I model). The reliability of the nodes was assessed with a bootstrap resampling procedure consisting of 1,000 replicates.

Animal experiments were performed to study the pathogenicity of the novel recombinant strain. This study was approved by the Jiangsu Academy of Agricultural Sciences Experimental Animal Ethics Committee (approval number: NKYVET 2023-0105). RHDV-seronegative 8-week-old New Zealand rabbits were randomly divided into two groups (*n* = 10/group). Rabbits in each group were injected subcutaneously with 2 × 10^4^ hemagglutination units (HAU) [18, 19] of novel SC2020/1016-02 strain or with the original RHDV2 SC2020/0401 control. At 12, 18, 24, 36, 48, 68, and 84 hr postinfection (hpi), blood samples of three rabbits from both groups were collected, and RHDV vp60 gene expression analysis was performed by quantitative RT-PCR using a real-time SYBR master mix (TaKaRa, Dalian, China) and a Light Cycler 480 II system (Roche, Basel, Switzerland). The primers (RHDV2-Fwd, 5′-TGGAACTTGGCTTGAGTGTTGA-3′ and RHDV2-Rev, 5′-ACAAGCGTGCTTGTGGACGG-3′) used for RT-qPCR have been described previously [20]. The expression of RHDV was normalized to that of the *β*-actin, and the results are presented as fold induction relative to 12 hpi blood samples of SC2020/0401 infected animals. Liver tissues were collected from rabbits at the moribund stage and negative control (0 hpi) (two rabbits per group), fixed in 4% paraformaldehyde, embedded in paraffin, and sectioned (4 *μ*m thick) for detection of liver injury using hematoxylin and eosin staining. The five remaining animals in each group were left for observation, and the dying rabbits were euthanized for the purposes of survival calculation.

## 3. Results and Discussion

The full-length genome sequences of hemagglutination-positive samples were obtained. A total of four isolates (SC2020/1016-02, SC2020/1025, JS2021/0125, and GX2021/0421; GenBank accession numbers: ON638912, ON638914, ON638915, and ON638913, respectively) collected from three different provinces (Sichuan, Jiangsu, and Guangxi provinces) in October 2020 to April 2021 were further analyzed. The four isolates belonging to the recombinant strain were similar, with a total of 81 nucleotide variations among the analyzed viruses. Specifically, 23 of these nucleotide variations altered amino acid sequences: 10 altered nucleotide sites located in nonstructural genes (124, 287, 349, 446, 1114, 3539, 4379, 4381, 4387, and 4403) and 13 altered nucleotide sites located in structural genes (5462, 5759, 5768, 5776, 5809, 5812, 6191, 6213, 6257, 6269, 6446, 6923, and 7374). Additionally, a Web BLAST search revealed that the strain shared less than 88.8% nucleotide sequence identity with other viruses. Interestingly, the nucleotide sequence of the structural protein genes VP60 and VP10 of the new strain was more than 99.0% identical to that of the Chinese GI.2 strain SC2020/0401, whereas the nucleotide sequence of the nonstructural protein domains was less than 85.0% identical. BLAST analysis showed that the nonstructural proteins of the novel strain exhibited the highest nucleotide and protein sequence identity (approximately 88.8% and 97.8%, respectively) with the strain JS-NATF2, which has been previously identified in seemingly healthy wild rabbits in China by meta-transcriptomic analysis [21].

The four novel isolates were subsequently aligned with publicly available GI.1–GI.4 sequences, and recombination events were screened using RDP. A single recombination breakpoint was detected with strong statistical evidence (*p*-values < 0.01) at the boundary of the nonstructural/structural parts (RdRp/VP60) at nucleotide position 5,301 in all four isolates, using six methods (RDP, GENECONV, BootScan, MaxChi, Chimaera, and 3Seq) implemented in RDP (Table 1). For the majority of the GI.1–GI.4 strains, the GI.2 strain SC2020/0401 was the most likely donor for the structural part, while the strain JS-NATF2 was identified as the most likely donor for the nonstructural part. Thus, according to RDP, the new strains investigated in this study were GI.2 recombinants. SimPlot analysis also showed that the 3′ end of the genome of the new variant SC2020/1016-02 was similar to that of SC2020/0401, whereas the nonstructural part was highly similar to JS-NATF2, with high nucleotide and protein sequence identity (between 85.4% and 92.7% and between 93.7% and 98.9%, respectively) to the seven nonstructural proteins (Figure 1).

**Figure 2:**
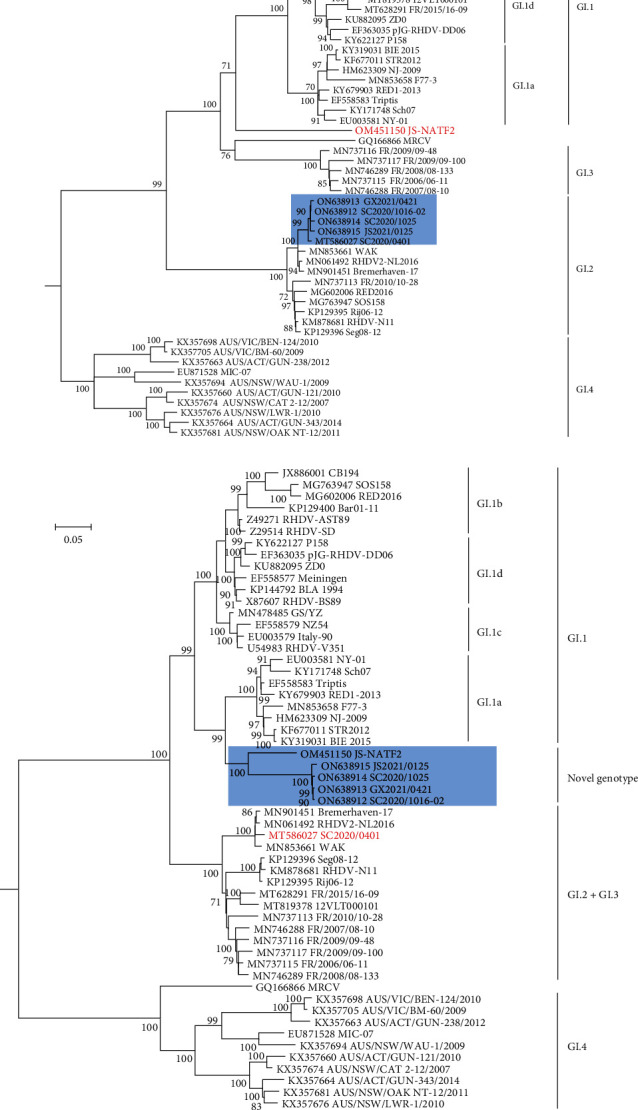
Maximum-likelihood (ML) phylogenetic trees for the genome regions defined by the recombination analysis. Bootstrap probability values above 70% with 1,000 replicates are indicated at the nodes. (a) ML tree based on the structural part (nucleotides 5,305–7,378; nucleotide substitution model: general time reversible model with a gamma distribution and invariant sites (GTR + G + I)). The group, including the new isolates obtained in this study and GI.2 strain SC2020/0401, are shaded in *blue*. The unclassified strain JS-NATF2 is marked in red. (b) ML tree based on the nonstructural part (nucleotides 10–5,304; nucleotide substitution model GTR + G + I). The group, including the new isolates obtained in this study and the unclassified strain JS-NATF2, is shaded in *blue*. The GI.2 strain SC2020/0401 is marked in *red*. GenBank accession numbers of the sequences are indicated in the taxon names. The European brown hare syndrome virus (EBHSV) strain GD (Z69620) was used as the outgroup to root the tree.

Maximum-likelihood phylogenetic trees inferred using MEGA 7 based on the nonstructural genes (nucleotide positions 10–5,304) and structural genes (VP60 and VP10; nucleotide positions 5,305–7,378) were employed to determine the evolutionary history of the new isolates [8]. As shown in the phylogenetic tree of the genes encoding the structural proteins, the new isolates clustered with SC2020/0401 (shaded blue) and other GI.2 strains (Figure 2(a)). Importantly, the construction of the phylogenetic tree of the nonstructural protein sequences revealed that the new isolates were clustered with unclassified JS-NATF2 strain in a well-supported group (bootstrap value of 100; shaded blue, Figure 2(b)) close to the GI.1a strains. Interestingly, the structural genes of JS-NATF2 formed a single branch that fell between the pathogenic GI.1 strains and the group, including European nonpathogenic rabbit caliciviruses (GI.3) and the weakly pathogenic Michigan rabbit calicivirus (MRCV) (red, Figure 2(a)). Therefore, phylogenetic analysis further confirmed the occurrence of recombination in the new isolates.

**Figure 3:**
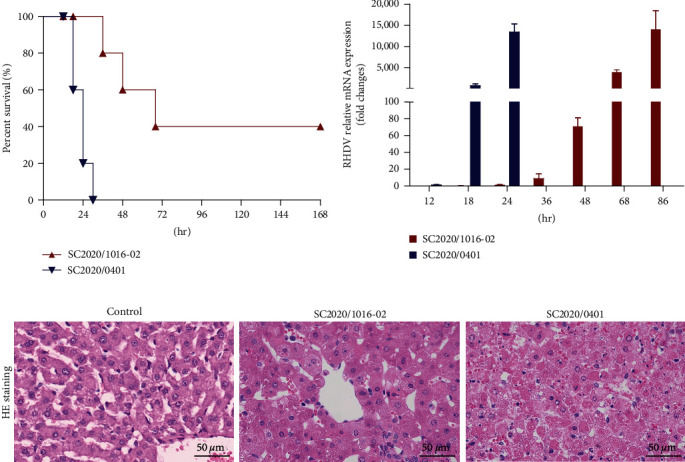
Pathogenicity of novel recombinant strains compared with the original strain of fatal rabbit hemorrhagic disease virus type 2 (RHDV2). (a) Survival of rabbits infected with SC2020/1016-02 and SC2020/0401. All rabbits were clinically examined daily for 7 days. (b) RHDV relative mRNA expression levels were detected. (c) Liver tissues were collected from rabbits at the moribund stage and negative control. Hematoxylin and eosin (HE) staining was used for histopathological evaluations (magnification, ×400).

In the challenge study, the survival time and mortality rate differed between recombinant (SC2020/1016-02) and original (SC2020/0401) groups. The strain SC2020/1016-02 induced 60% death of injected rabbits, lower than that of the original RHDV2 strain SC2020/0401, which had a 100% mortality rate (Figure 3(a)). The mRNA level of RHDV was replicated in the liver in a time-dependent manner in the two groups. Compared with the original strain SC2020/0401, the recombinant strain SC2020/1016-02 had a longer replication time and slower replication speed, with almost the same viral load at the moribund stage of infection (Figure 3(b)). Pathological examinations showed that liver lesions characterized by necrotic hepatocytes were more severe in SC2020/0401 infected rabbits than in SC2020/1016-02 infected rabbits and not observed in control rabbits (Figure 3(c)).

China is the largest global rabbit meat producer. Sichuan province is majorly involved in rabbit breeding and consumption, as well as in the trade of rabbit products with other provinces [22]. Given the distance and geographic barriers between Sichuan province and Jiangsu and Guangxi provinces (approximately 1,500 and 1,000 km, respectively), it is likely that anthropogenic factors were responsible for the introduction of novel strains in other provinces. Indeed, Sichuan province is the area with the highest rabbit meat consumption in China [22]. Also, the live-rabbit trade bustles between Sichuan and other breeding areas, which facilitates virus transmission through people and vehicles. The detection of a novel recombinant strain from three different provinces over a 6-month period suggests the circulation of the novel GI.2 strain, which might influence the genetic profile and molecular evolution of RHDV in China. Indeed, more diverse viral populations can result in more virulent infections and improved adaption to new hosts [23]. However, the level of competition of the novel recombinant GI.2 strain against the original GI.2 strain remains unknown.

The emergence of pathogenic viruses is of great concern. Recombination has been recognized as a major force driving RNA virus evolution associated with the emergence of disease outbreaks or host range expansion [24, 25]. Our study is the first to report the circulation of a novel recombinant GI.2 strain in China, with the GI.2 strain serving as the major donor for the structural part and an unclassified lagovirus genotype contributing to the nonstructural part. As the nonstructural proteins donor strain JS-NATF2 circulates in seemingly healthy wild rabbits in China, it might be a nonpathogenic or weakly pathogenic rabbit calicivirus similar to GI.3 or MRCV, and we can hypothesize that the novel recombinant GI.2 strain described in this study emerged in China. Our study revealed that recombination events resulting in the emergence of novel pathogenic strains occurred within 6 months to 1 year after the initial detection of RHDV2 in China, illustrating the high capacity of recombination within lagoviruses.

New strains having distinct features seem to arise quickly due to recombination, which may act as a survival strategy and confer additional epidemiological benefits. Indeed, there is mounting evidence for recombination in caliciviruses, particularly at the 5′ end of the structural genes [26–28]. A previous study proposed a mechanism for recombination in norovirus. Norovirus recombination occurs when the virus replicase initiates positive-strand synthesis at the 3′ end of the negative strand, loses the processivity at the stem-loop position, and then, the template switches to negative genomic or subgenomic RNA from the coinfecting virus [26]. Considering that RHDV has a similar genomic pattern to that of noroviruses, RHDV might use the same recombination mechanism to generate new recombinant viruses. The phylogenetic analysis of the new virus showed that the pathogenic GI.2 strain served as the major donor for the structural part and that the unclassified nonpathogenic strain contributed to the nonstructural part, indicating that the structural genes of RHDV might play an important role in pathogenicity [8]. Hence, analysis of full genomic sequences may improve our understanding of the evolutionary history and genetic relationships of lagoviruses. In addition, the pathogenicity of recombinant RHDV2 also showed some competitive advantages over the original RHDV2. The new strain showed a longer infected time and lower mortality rate, but the viral load was almost the same at the moribund stage of infection with the original strain. This might have resulted in high virus contamination in the environment and a greater chance of infecting other animals via recombinant RHDV2 transmission. Moreover, longer infection times might increase the chance of cross-region infection via live-rabbit trade. For this reason, it is speculated that the new recombinant strain might have competitive advantages in viral transmission over the original RHDV2. Meanwhile, more epidemiological data are needed to describe the evolutionary advantage of the new recombinant RHDV2 over the original pathogenic RHDV2. The dominance of emerging RHDV2 variants cannot be attributed to enhanced fitness without evidence of replacement [29]. Thus, ongoing detection and monitoring of RHDV2 are important to understand whether the recombinant strain is becoming dominant over the original RHDV2. Furthermore, as RHDV2 causes fatal hepatitis in both rabbits and hares, GI.2 and related recombinants may not only affect the rabbit industry but also the ecosystem. Considering the lack of vaccines against RHDV2, ongoing surveillance, and vaccine development are imperative for the control of the disease induced by RHDV2 and RHDV2 recombinants in China.

## Figures and Tables

**Table 1:** Results of the recombination analysis for novel RHDV2 strains using RDP software.

Strains (collect province)	Most likely donor strain		Break point^c^	Methods and average *p*-values					
	NSP^a^	SP^b^		RDP	GENECONV	BootScan	MaxChi	Chimaera	3Seq
SC2020/1016-02 (Sichuan)	JS-NATF2 (unclassified genotype)	SC2020/0401 (GI.2)	5,301 (5,251–5,420)	6.606 × 10^−111^	1.502 × 10^−101^	4.241 × 10^−114^	2.361 × 10^−4 0^	1.230 × 10^−28^	4.170 × 10^−48^
SC2020/1025 (Sichuan)			5,301 (5,248–5,405)	1.607 × 10^−103^	9.300 × 10^−98^	3.104 × 10^−107^	2.620 × 10^−39^	1.351 × 10^−27^	1.142 × 10^−49^
JS2021/0125 (Jiangsu)			5,301 (5,248–5,405)	6.049 × 10^−107^	9.419 × 10^−100^	8.189 × 10^−108^	4.020 × 10^−4 0^	1.026 × 10^−28^	2.962 × 10^−50^
GX2021/0421 (Guangxi)			5,301 (5,251–5,420)	4.508 × 10^−106^	2.647 × 10^−98^	2.202 × 10^−109^	1.228 × 10^−39^	9.931 × 10^−28^	5.187 × 10^−48^

*Notes*: ^a^Nonstructural proteins. ^b^Structural proteins. ^c^99% confidence interval is indicated.

## Data Availability

All virus genome sequences have been submitted to the National Center for Biotechnology Information (NCBI). All other materials are available from the author of the correspondence.
